# Integrative proteomic and transcriptomic analysis provides evidence for TrkB (NTRK2) as a therapeutic target in combination with tyrosine kinase inhibitors for non-small cell lung cancer

**DOI:** 10.18632/oncotarget.24361

**Published:** 2018-01-30

**Authors:** Daniel Richard Gomez, Lauren Averett Byers, Monique Nilsson, Lixia Diao, Jing Wang, Lerong Li, Pan Tong, Mia Hofstad, Babita Saigal, Ignacio Wistuba, Neda Kalhor, Stephen Swisher, Youhong Fan, Waun Ki Hong, Milind Suraokar, Carmen Behrens, Cesar Moran, John Victor Heymach

**Affiliations:** ^1^ Department of Radiation Oncology, University of Texas MD Anderson Cancer Center, Houston, TX, USA; ^2^ Department of Thoracic/Head and Neck Medical Oncology, University of Texas Anderson Cancer Center, Houston, TX, USA; ^3^ Department of Bioinformatics and Computational Biology, Division of Quantitative Sciences, University of Texas MD Anderson Cancer Center, Houston, TX, USA; ^4^ Department of Translational Molecular Pathology, Division of Pathology and Laboratory Medicine, University of Texas MD Anderson Cancer Center, Houston, TX, USA; ^5^ Department of Pathology Administration, Division of Pathology and Laboratory Medicine, University of Texas MD Anderson Cancer Center, Houston, TX, USA; ^6^ Department of Thoracic and Cardiovascular Surgery, Division of Surgery, University of Texas MD Anderson Cancer Center, Houston, TX, USA

**Keywords:** TrkB, squamous cell carcinoma, lung cancer, proteomics

## Abstract

While several molecular targets have been identified for adenocarcinoma (ACA) of the lung, similar drivers with squamous cell carcinoma (SCC) are sparse. We compared signaling pathways and potential therapeutic targets in lung SCC and ACA tumors using reverse phase proteomic arrays (RPPA) from two independent cohorts of resected early stage NSCLC patients: a testing set using an MDACC cohort (N=140) and a validation set using the Cancer Genome Atlas (TCGA) cohorts. We identified multiple potentially targetable proteins upregulated in SCC, including NRF2, Keap1, PARP, TrkB, and Chk2. Of these potential targets, we found that TrkB also had significant increases in gene expression in SCC as compared to adenocarcinoma. Thus, we next validated the upregulation of TrkB both *in vitro* and *in vivo* and found that it was constitutively expressed at high levels in a subset of SCC cell lines. Furthermore, we found that TrkB inhibition suppressed tumor growth, invasiveness and sensitized SCC cells to tyrosine kinase EGFR inhibition in a cell-specific manner.

## INTRODUCTION

Over the past decade, there have been major advancements in the targeted treatment of non-small cell lung cancer (NSCLC), which have improved outcomes in a subset of patients. However, most of this progress has been made in lung adenocarcinoma, through the identification of mutations of the epidermal growth factor receptor (EGFR), EML4-ALK fusions, and other driver alterations that are highly sensitive to targeted drug therapy. In contrast, however, there have been few therapeutics targeted for SCC. Inhibitors of the immune checkpoint factor PD-1 have recently been shown to prolong survival in platinum-refractory SCC [1, 2]. EGFR TKIs also provide benefit in SCC [3, 4], although response rates are low and benefits are typically short-lived prior to the emergence of drug resistance [5, 6]. The EGFR monoclonal antibodies cetuximab and necitumumab have demonstrated benefit in SCC patients, prolonging survival by 1-2 months overall [7, 8]. These studies suggest that intrinsic resistance to EGFR inhibitors emerges rapidly and limits the long term effectiveness of these agents. There is thus an unmet need both to identify agents that may impede resistance to EGFR inhibitors in adenocarcinoma of the lung, as well as to to identify new therapeutic targets for SCC.

Our group has previously utilized reverse phase protein array (RPPA) to identify altered pathways in NSCLC and proteins associated with recurrence and survival outcomes [9, 10]; new therapeutic targets (e.g., PARP in SCLC) [11, 12]; and markers associated with drug resistance [13]. For example, proteomic profiling of small cell lung cancer (SCLC) identified PARP1 as a therapeutic target [11, 14], despite the fact that PARP1 protein overexpression in SCLC is not associated with any known alterations at the DNA level (mutation, copy number gains/losses, fusions). This finding is now supported by clinical studies demonstrating single agent activity for PARP inhibition in refractory SCLC [15]. These studies illustrate that proteomic analyses can yield unique and often complementary data to that offered with genomic analyses. In addition, a recent presentation at the American Society of Clinical Oncology demonstrated that larotrectinib, a small-molecule pan-TRK inhibitor, was associated with durable antitumor activity in patients with TRK fusions and a wide variety of malignancies, including lung cancer [16]. In this light, there is renewed interest in understanding which patient subsets may benefit from this therapy.

In the current study, we utilized RPPA analysis to detect potential therapeutic targets expressed at higher levels in previously untreated [treatment naïve], surgically resected SCC tumors of the lung as compared to resected non-SCC NSCLC (predominantly adenocarcinoma) in a cohort of 140 NSCLC patients from our institution. Findings were then validated in a large, independent cohort of lung tumors from The Cancer Genome Atlas (TCGA) (195 SCC, 181 adenocarcinomas). We then used gene expression data to support the RPPA findings in select proteins, both with the PROSPECT and TCGA databases. The top candidate drug targets identified by this analysis were then tested utilizing appropriate targeted agents to determine their activity in SCC cell lines models. Finally, due to the finding of TrkB as overexpressed in SCC, we performed *in vitro* and *in vivo* validation of the role of this factor in tumor progression, also focusing on its interaction with the EGFR pathway.

## RESULTS

### Institutional tissue specimens and patient characteristics

The prospective institutional database that was utilized was that of the Profiling of Resistance Patterns and Oncogenic Signaling Pathways in Evaluation of Cancers of the Thorax (PROSPECT), which was developed in 2006 with the purpose of investigating novel molecular profiling mechanisms of therapeutic resistance, and in turn generating rational therapeutic strategies for overcoming resistance [17]. Supplementary Table 1 depicts patient characteristics in the PROSPECT dataset. Twenty four percent of patients had SCC histology (n=34), while 76% had non-SCC (n=106). Fifty eight percent of patients were male, and 91% were smokers. The stage distribution was: I=56% (n=78); II=18% (n=24); III=26% (n=36); IV=<1% (n=1) (Supplementary Table 1). Protein expression levels for 127 total and phosphoproteins were compared between SCC and non-SCC by t-test. Supplementary Table 1 also provides a comparison of PROSPECT with TCGA, both SCC and non-SCC.

### Proteomic profiling identifies key differences in protein expression in lung SCC

**Figure 1:**
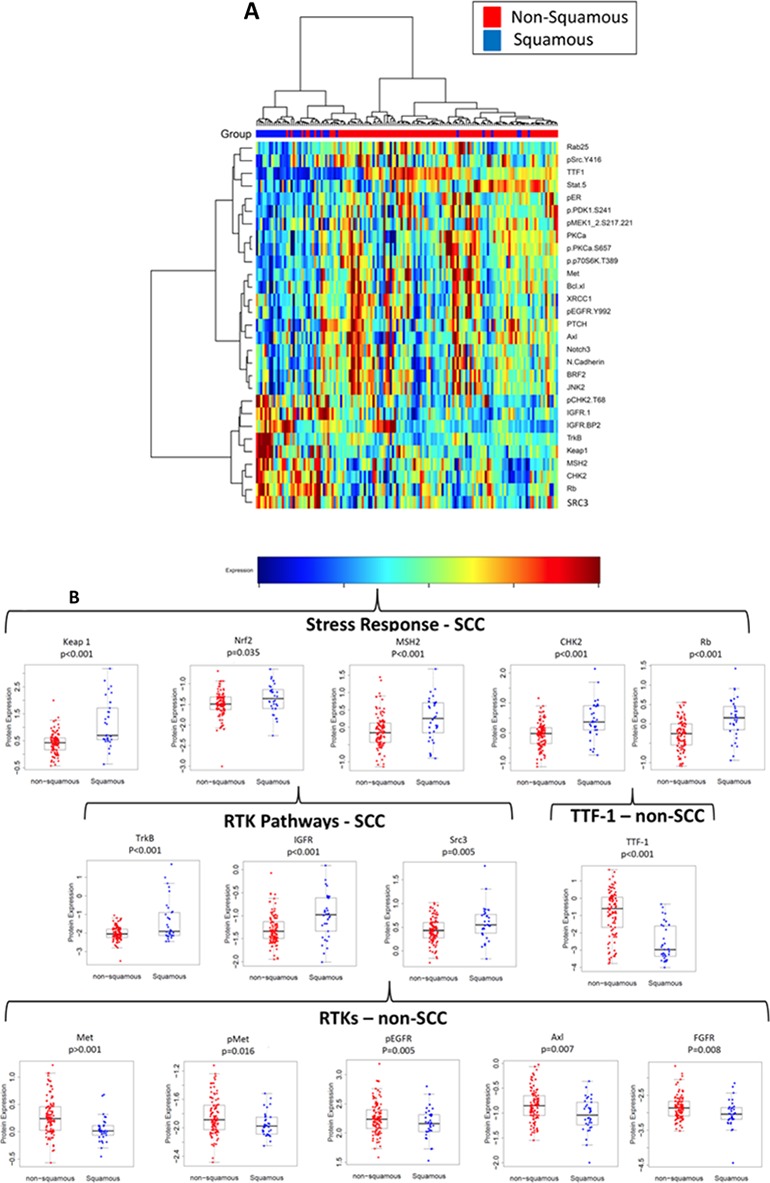
Differences in protein expression of squamous cell carcinoma (SCC) vs. non-SCC **(A)** hierarchical clustering of proteins strongly associated with SCC or non-SCC histology (top 29 hits based on p<0.05), **(B)** Proteins involved in the stress response (Keap1, MSH2, CHK2) were increased with SCC, and TTF-1 was elevated in non-SCC. Changes in proteins involved in RTK pathways varied, with some increasing and others decreasing.

Figure 1A demonstrates hierarchical clustering of the top 29 proteins (p<0.05) that were differentially expressed between these histologic subgroups in the PROSPECT cohort. To adjust for multiple comparisons, we applied a beta-uniform mixture (BUM) to model the resulting p-values computed from the test statistic, and a false detection rate (FDR) of 1%. Through this analysis, we determined that several proteins related to the stress response and/or DNA repair were relatively higher in SCC, including Keap1 (p<0.001), Nrf2 (p=0.035), CHK2 (p<0.001), pCHK2 (p<0.001), Rb (p<0.001), cleaved PARP (p=0.026), and MSH2 (p<0.001) (Table 1 and Figure 1B). Furthermore, while most of the increased expression of RTKs was in adenocarcinoma, two RTKs were expressed at relatively higher levels in SCC: the neurotrophic tyrosine kinase receptor, type 2, TrkB (p<0.001), which is involved in neuronal differentiation and cell survival (Table 1, Figure 1B) and insulin-like growth factor receptor (IGFR, p<0.001). Furthermore, the steroid receptor coactivator-3 (Src 3, also termed AIB1), a member of the p160 src family which has been shown to regulate the expression of IGF-1 [18, 19], was also upregulated in SCC. Supplementary Table 2 demonstrates the full list of proteins statistically significantly associated with histology (p<0.05) in the PROSPECT database, listed by strength of association.

**Table 1:** Selected targetable proteins differentially expressed between SCC and non-SCC (p<0.05)

*Protein Marker*	*Difference (fold-change)*	*T Score*	*p-value*
***Increased in SCC***			
***Stress Response***			
*Keap1*	*1.64*	*6.79*	*<0.001*
*Nrf2*	*1.10*	*2.51*	*0.035*
*CHK2*	*1.41*	*5.16*	*<0.001*
*pCHK2*	*1.28*	*5.13*	*<0.001*
*Rb*	*1.34*	*5.14*	*<0.001*
*MSH2*	*1.28*	*3.56*	*<0.001*
*PARP*	*1.37*	*2.25*	*0.026*
***Receptor Tyrosine Kinase (RTK)***			
*IGF1R*	*1.23*	*4.14*	*<0.001*
*TrkB*	*1.56*	*5.34*	*<0.001*
***Downstream Regulators of RTKs***			
*Src3 (IGF-1)*	*1.12*	*2.84*	*0.005*
***Decreased in SCC***			
***TTF-1***	*-3.41*	*-7.17*	*<0.001*
***RTKs***			
*pEGFR*	*-1.11*	*-2.82*	*0.005*
*Met*	*-1.17*	*-3.71*	*<0.001*
*pMet*	*-1.08*	*-2.44*	*0.016*
*Axl*	*-1.13*	*-2.75*	*0.007*
*FGFR*	*-1.14*	*-2.67*	*0.008*
***Downstream Regulators of***			
***RTKs***			
*pMek*	*-1.14*	*-4.43*	*<0.001*
*Rab25*	*-1.28*	*-3.76*	*<0.001*
*p70S6K*	*-1.15*	*-3.66*	*<0.001*
*PKCα*	*-1.26*	*-3.95*	*<0.001*
*JNK2*	*-1.12*	*-3.21*	*0.002*
*p90RSK*	*-1.09*	*-2.09*	*0.038*
*mTOR*	*-1.08*	*-2.03*	*0.044*

In contrast, TTF-1, a sensitive marker for adenocarcinomas of lung origin, was substantially lower in the SCC cohort (p<0.001, 3.4-fold lower in SCC), as were several classical receptor tyrosine kinases (RTKs), including pEGFR (p=0.005), Met (p<0.001), pMet (p=0.016), Axl (p=0.007), and FGFR1 (p=0.008). In addition, several proteins downstream of these RTKs demonstrated lower expression in SCC, including pMek (p<0.001), p70S6K (p<0.001), JNK2 (p=0.002), p90RSK (p=0.038), and mTOR (p=0.044). Additional proteins related to the EGFR pathway that were downregulated in SCC were PKCα (previously shown to mediate EGFR expression [20] and take part in mTOR regulation by EGFR [21]) and Rab25 which plays a role in EGFR trafficking through receptor internalization/recycling [22] and can activate the PI3K/AKT pathway through phosphorylation of AKT [23].

### Analysis of protein expression in TCGA validates increased protein expression of key signaling pathways in NSCLC, including chk2 and SRC-3

To validate differences in protein expression, we then compared expression levels in an independent set of SCC and adenocarcinomas from the TCGA (patient characteristics in Supplementary Table 1). Protein expression levels for 227 total and phosphoproteins were compared between SCC and non-SCC by t-test, with 92 proteins overlapping between both groups/tested in both groups. Hierarchical clustering of the TCGA samples shows a clear separation between lung squamous and lung adenocarcinomas tumors, based on distinct protein expression profiles (Supplementary Figure 1A). Among those proteins measured in both patient cohorts, several differentially expressed in PROSPECT also showed similar differences in the TCGA cohort. Specifically, of the 53 proteins that were statistically significantly increased in the PROSPECT database (p<0.05), 23 of these showed the same trend in the TCGA. Four proteins had a trend in the same direction that was not statistically significant (p≤0.20), 3 had no trend in either direction (p>0.20), and 7 had the opposite effect as was observed in PROSPECT (p<0.05), as demonstrated in Supplementary Table 2. Notable proteins that were also increased in SCC tumors in the TCGA database included Keap1 (p<0.001), Cyclin B1 (p<0.001), MSH2 (p<0.001), and Nrf2 (p<0.001). It is worth noting that TrkB proteomic data was not available from the TCGA. To further illustrate these differences, Supplementary Figure 1B shows the TCGA validation of the subset of the top 29 proteins that varied by histology in PROSPECT that were also tested in TCGA. Again, it is evident that the differences in SCC vs. non-SCC were present in both databases. Finally, Supplementary Figure 1C depicts dot plots of selected histologic associations in TCGA, specifically a reduction of TTF-1 in SCC (p<0.001) and increased protein expression of Chk2 (p<0.001), pChk2 (p<0.001), and Src 3 (p=0.014).

Based on the PROSPECT and TCGA analyses, we chose to further explore gene expression and drug sensitivity data on TrkB, Chk2, and NCOA3, as they met the following criteria: 1) higher expression in SCC; 2) high levels of associations with histology (SCC subtype) in both clinical datasets (when available); 3) directly targetable, but with limited clinical evidence pertaining to the appropriate targeted agent; and 4) prior evidence demonstrating that the targeting the protein or pathway could affect outcome in NSCLC. We omitted other proteins that were found to have differing expression based on histology that did not meet all of the criteria. For example Rb and MSH2, while differentially regulated, are not readily targetable. In contrast, while IGFR meets three of these criteria, IGF1R antagonists have been studied in several solid tumors [24, 25] and did not demonstrate benefit in phase III testing in NSCLC, and were therefore not investigated further in this study [26].

### Gene expression profiling demonstrates increased expression of NTRK2 and CHEK2 in SCC tumors

We next sought to validate potential SCC targets with gene expression profiling of three proteins, as described above: 1) *NTRK2* (the gene coding for TrkB protein), 2) *CHEK2* (coding for CHK2), and 3) *NCOA3* (coding for SRC-3). TrkB has been shown to be associated with EMT transition and poor prognosis in lung cancer [27–29], as well as increased aggressiveness in other malignancies [30, 31]. CHK2 is a DNA repair protein that, when expressed at lower levels, has been shown to be correlated with worse survival in SCC lung cancer [32]. Furthermore, Chk inhibitors have been tested (e.g. AZD7762) in phase I trials of advanced solid tumors [33]. SRC-3 has histone acetyltransferase activity and has been associated with lung cancer cell invasion and poorer survival. It has previously been implicated as a potential target in lung cancer and has been shown to be amplified in breast and ovarian cancer [34].

**Figure 2:**
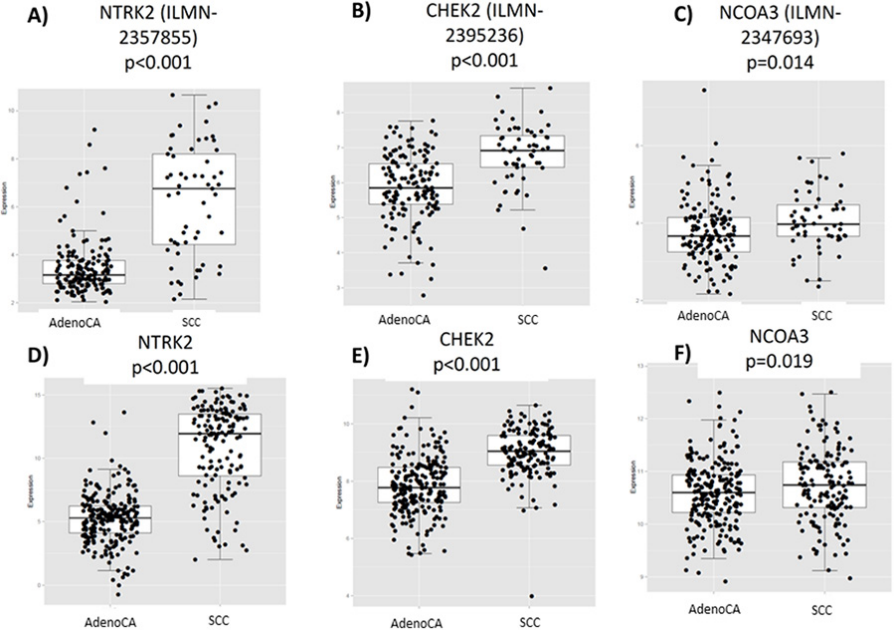
Differences in gene expression by histology in the PROSPECT and TCGA databases mRNA levels of NTRK2, CHEK2, and NCOA3 are higher in SCC tumors in PROSPECT **(A-C)** and TCGA **(D-F)** datasets.

By analyzing separate probes of *NTRK2, CHEK2*, and *NCOA3*, we found that out of all probes tested, *NTRK2* and *CHEK2* were associated with the most substantially increased gene expression in SCC (Figure 2). We also found that when comparing profiles of lung cancer SCC, head/neck SCC, and lung cancer adenocarcinoma, expression profiles were more similar between lung and head/neck SCC than with lung adenocarcinoma and lung SCC. Supplementary Figure 2 demonstrates the similarity between lung cancer and head/neck SCC in NTRK2. Supplementary Figures 3-5 depict the relationship of every probe tested in the PROSPECT database of NTRK2, CHEK2, and NCOA3, respectively. We could not find substantial differences between the two probes utilized for NCOA3 (Supplementary Figure 6), but for CHEK2, the probe that was correlated with SCC histology was the more reliable of the two, thereby strengthening the relationship between increased expression of this protein and SCC histology (Supplementary Figure 7).

### *In vitro* and *in vivo* validation of TrkB as a therapeutic target

Given that TrkB was overexpressed at the protein and mRNA level in squamous lung cancers and has previously been implicated in disease progression in lung cancer and other malignancies, we then hypothesized that TrkB may represent a therapeutic target in squamous-type NSCLC. We tested this preclinically using multiple approaches. First, we assessed the expression of TrkB in both SCC and adenocarcinoma cell lines. Second, we added AZD7451, a TRK inhibitor, to both SCC and non-SCC cell lines with or without BDNF, the ligand for TrkB, to determine if the addition of this ligand could reverse the inhibitory effect. Third, we explored the effect of stimulation and inhibition of TrkB on cell migration, based on prior data in other malignancies suggesting that there may be crosstalk between EGFR and TrkB signaling [35]. Finally, based on this same premise, we combined EGFR inhibitors with TrkB tyrosine kinase inhibitors to determine if there was a synergistic effect of targeting these pathways concurrently.

### *In vitro* validation of TrkB as a target in SCC shows increased expression in SCC head and neck cell lines

**Figure 3:**
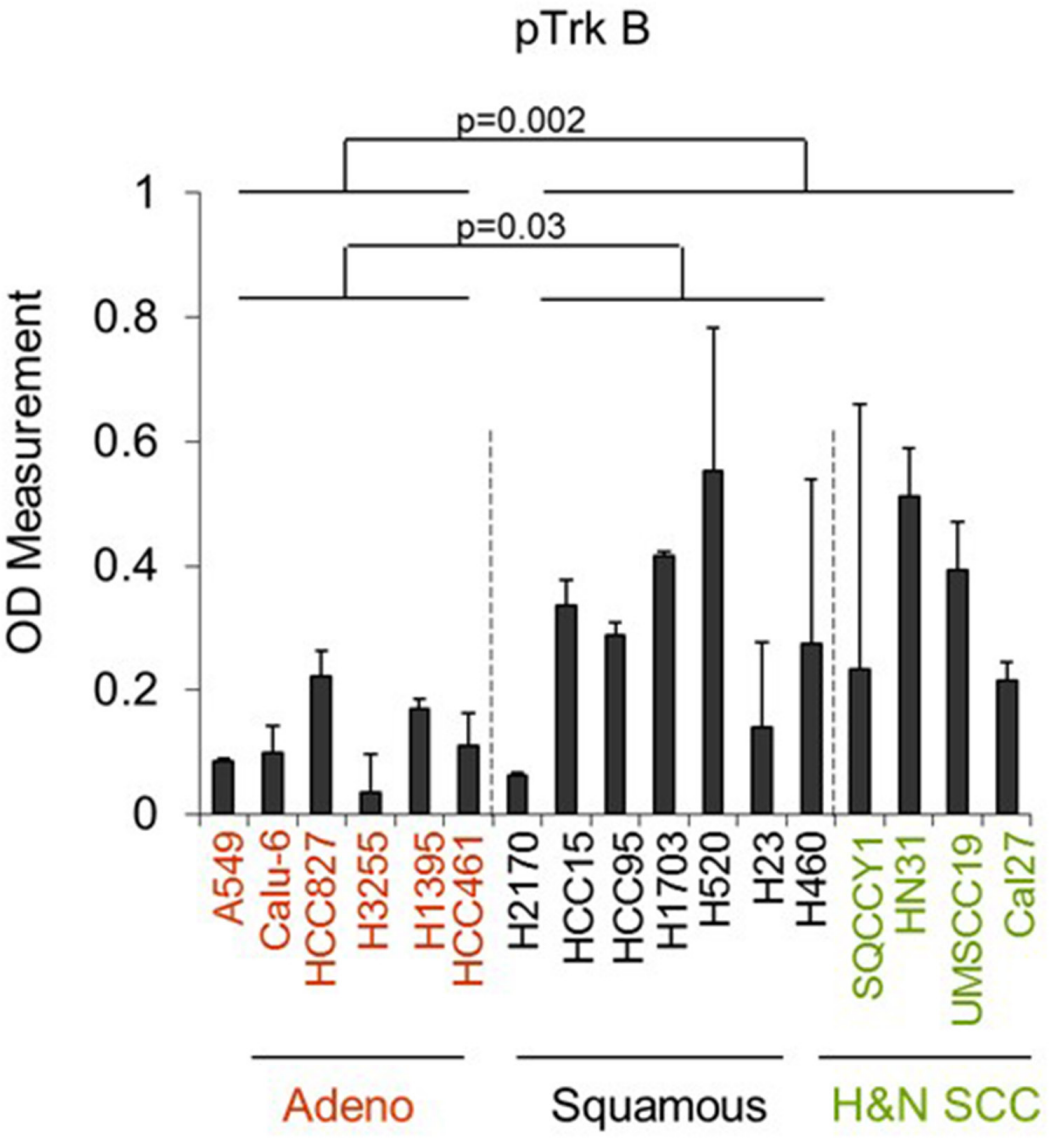
Phosphorylated TrkB levels are elevated in SCC and head and neck SCC compared with lung adenocarcinoma cell lines

**Figure 4:**
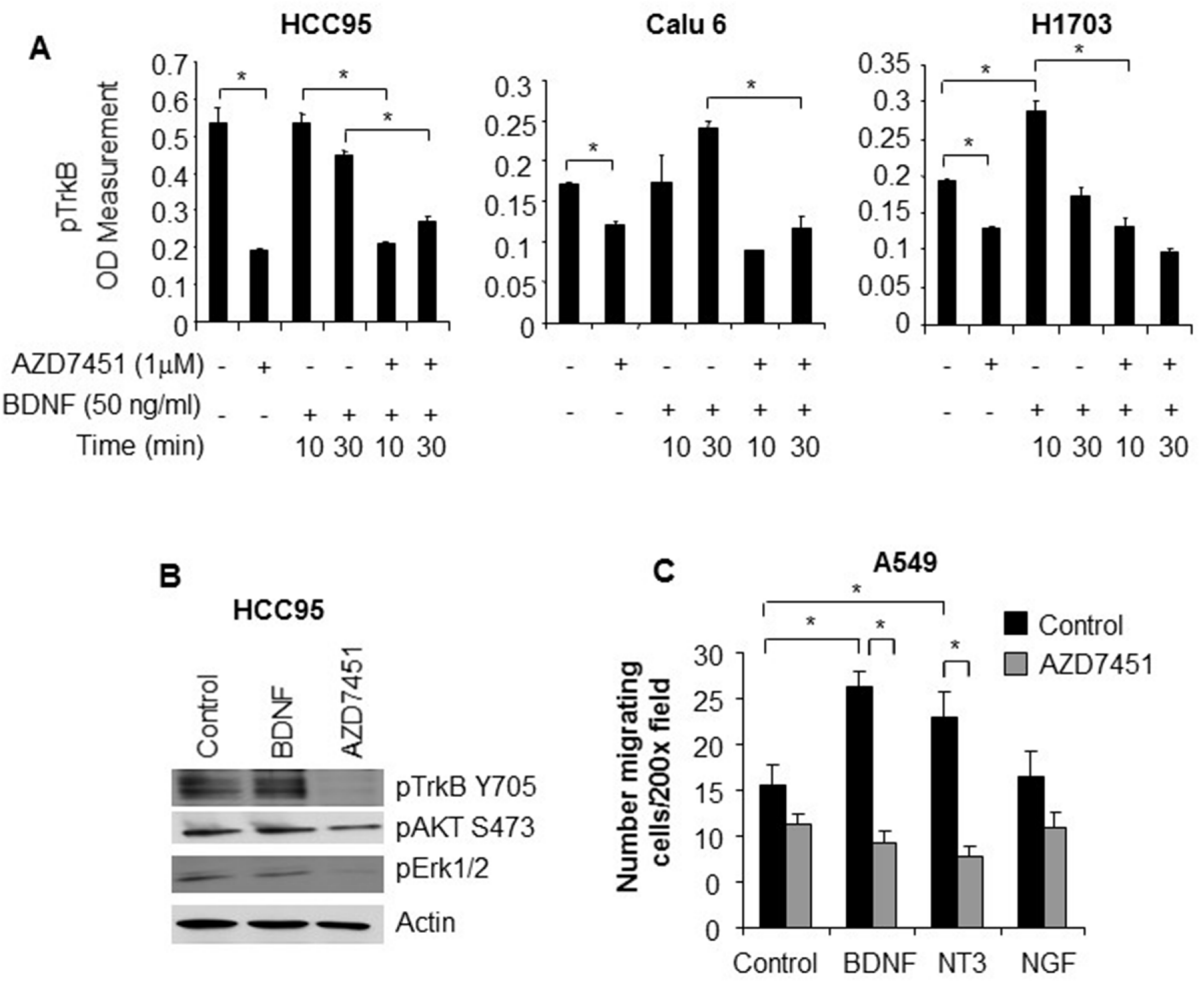
**(A)** TrkB ELISA in SCC (HCC 95), adenocarcinoma (Calu 6), and adenosquamous (H1703) cell lines showing high constitutive levels of TrkB in the HCC 95 cell line (high levels with control media only). In all three cell lines, adding a TrkB inhibitor, AZD7451 reduces TrkB signaling and in the cell lines with an adenocarcinoma component (Calu 6 and H1703), adding BDNF causes an increase in TrkB expression. **(B)** Western blot showing constitutive TrkB expression in HCC95 cell lines. The addition of BDNF has no effect, but adding AZD7451 reduces expression of both TrkB and its downstream target pERK 1/2. **(C)** Effect of BDNF on cell migration (a mechanism of tumor progression) showing that BDNF induces more cell migration when activating TrkB than NGF on TrkA and NT3 on TrkC. Migration is inhibited by AZD7451 with all three ligands, suggesting crosstalk between Trk receptors. The ^*^ denotes statistical significance.

We evaluated baseline levels of phosphorylated TrkB across a panel of adenocarcinoma and SCC cell lines by ELISA assay. We found that pTrkB expression was higher in both SCC and head/neck cell lines than in cell lines derived from adenocarcinoma of the lung. (Figure 3). We then treated NSCLC cell lines (HCC95=SCC, Calu 6=adenocarcinoma, H1703= adenosquamous) with BDNF alone or in the presence of the TrkB inhibitor AZD7451 and compared phopho-TrkB levels. The addition of AZD7451 significantly reduced the level of phospho-TrkB (p=0.04), whereas BDNF had little effect on the high baseline expression. In H1703, the adenosquamous cell line, there was lower baseline level of phospho-TrkB that could be both inhibited by AZD7451 (p=0.004) and increased with BDNF stimulation (p=0.02). Moreover, AZD7451 inhibited BNDF-induced TrkB phosphorylation (p=0.005). Similar results were observed with Calu6 cells. (Figure 4A). These results are further supported by Western blots of HCC95 cells, where treatment with BDNF did not change expression, but inhibition of the pathway with AZD7451 caused a reduction in both pTrkB and pERK 1/2, a downstream target of pTrkB (Figure 4B).

We also assessed the effects of addition of ligands to TrkA, TrkB, and TrkC on cell migration, under the hypothesis that TrkB is altering the invasive properties of malignant cells. Comparing NGF, BDNF, and NT3, the ligands of TrkA, TrkB, and TrkC, respectively, we found that BDNF stimulated the greatest increase in cellular migration (p<0.001), which then was inhibited by AZD7451 (p<0.001) (Figure 4C). This provides further support that the anti-invasive effects of AZD7451 are primarily due to its inhibition of TrkB although we cannot rule out the possibility that inhibition of other RTKs (e.g. TrkA and TrkC) contributes to the effects. We then performed the migration assay on three other cell lines: H23, H460, and A549. While A549 demonstrated increased migration with BDNF (p=0.0004), the change in H23 was not significant (p=0.37) and H460 was significant with the opposite trend (p=0.0003). These findings imply that the effect of TrkB on invasiveness is cell line specific.

### Combining EGFR inhibitors with TrkB tyrosine kinase inhibitors produces a cell-line specific synergistic effect *in vitro* and *in vitro*

**Figure 5:**
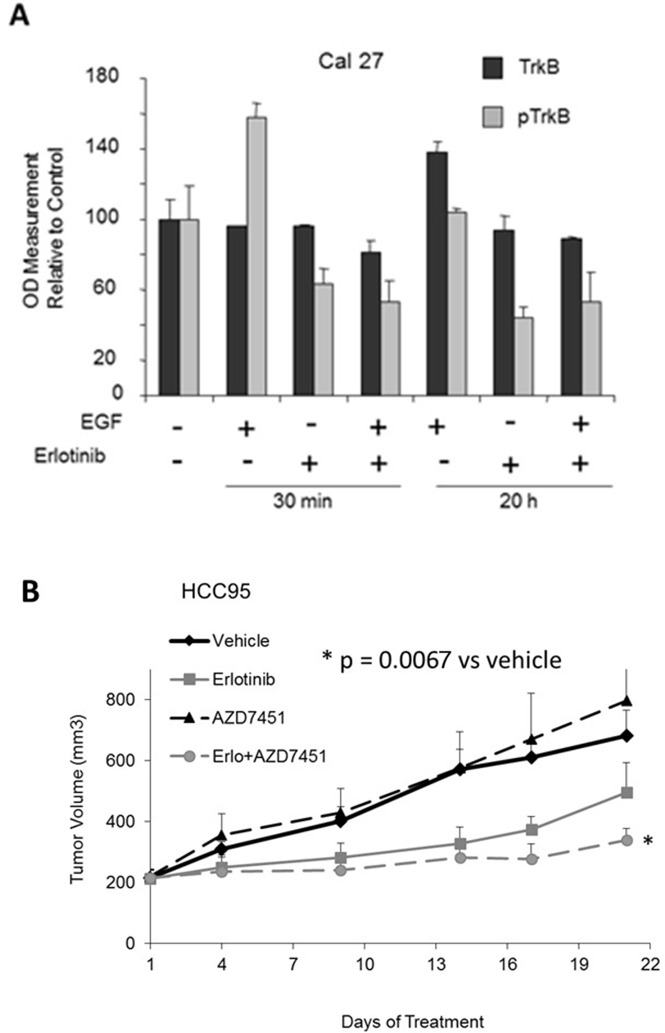
Crosstalk between EGFR and TrkB *in vitro* and *in vivo* **(A)** In Cal-27, a head and neck SCC cell line, adding EGF causes an increase in pTrkB, and the effect is inhibited by erlotinib at 30 minutes. At 20 hours, adding EGF causes an increase in TrkB, again inhibited by erlotinib. **(B)** *in vivo* analysis shows tumor volumes vs. treatment time with vehicle control, erlotinib, AZD7451, and erlotinib+AZD7451 for HCC95 cells. At day 21, only the combination therapy has significantly different tumor size compared to the vehicle control (p=0.0067). Both of these studies support the utilization of combined EGFR and TrkB inhibition as a therapeutic approach for SCC of the lung.

Given that AZD7451 inhibits TrkB activation and tumor cell migration, as well as the observed crosstalk between EGFR and TrkB in other malignancies [35], we then combined this agent with erlotinib to determine the effect on multiple SCC and adenocarcinoma cell lines. Indeed, both lung cancer and head and neck (H/N) cell lines were used, given prior pan-cancer analyses of the TCGA demonstrating consistencies in molecular profiles between lung and H/N SCC tumors [36–38] (Supplementary Figure 8). We found a synergistic effect in all adenocarcinoma cell lines except for one (Calu-27 was neutral). In SCC, we found that there was synergy in 2 cell lines, including one head and neck SCC, neutrality in one cell line (H520), and antagonism in 2 cell lines. However, it is notable that in the two cell lines in which antagonism was observed, the effect of AZD7451 alone was marked, thus likely making it more difficult to observe synergy with the combination regimen (Supplementary Table 3). Using Cal-27 cells, we next evaluated potential crosstalk between EGFR and TrkB. Cal-27 cells were serum starved for 24 hours and then treated with 50 ng/ml EGF alone or with 1M erlotinib for 30 minutes or 20 hours. Protein lysates were collected and phosphorylated TrkB levels were evaluated by ELISA assay. EGF treatment resulted in activation of TrkB, and this was attenuated by the addition of erlotinib, both at 30 minutes and 20 hours (Figure 5A). We then performed the same experiment in several other cell lines, including A549, H23, and H460. We did not observe crosstalk in any of these additional cell lines, again implying cell line specificity with this finding.

To evaluate the *in vivo* anti-tumor activity of AZD7451 alone and in combination with erlotinib, we tested several cell lines and ultimately utilized HCC95 because they readily formed xenografts in mice. We thus injected HCC95 cells subcutaneously into nude mice. Once tumors reached a volume of 200mm^3^, animals (n=5/group) were randomized to receive vehicle control, AZD7451 (20mg/kg), erlotinib (100 mg/kg), or the combination of AZD7451 plus erlotinib. Tumors were measured daily. Figure 5B demonstrates tumor volume size vs. treatment time, plotted as the mean +/− standard error of the mean (SEM). Overall treatment effect bordered on statistical significance (p=0.0889), and at day 21, only the combination therapy is significantly different compared to the vehicle control. We also compared treatment effect between the erlotinib and combination regimen, with the combination regimen bordering on statistical significance compared to erlotinib alone (p=0.0595).

## DISCUSSION

In this study, we examined protein and gene expression profiles in both SCC and non-SCC subtypes of NSCLC to determine if novel targets or therapeutic approaches could be identified. First, we found that multiple pathways varied by histology, with an increase in “stress response” proteins in SCC, particularly the oxidative stress pathway (e.g. NRF2) and DNA repair proteins, but lower levels of most RTKs. There were however, important exceptions to this observation, including enhanced expression of TrkB, a neurotrophic tyrosine kinase, and IGF-1R. Second, through a proteomic analysis of the PROSPECT and TCGA databases, we identified three novel targets that were upregulated in SCC: TrkB, CHK2, and SRC-3. We then validated the correlation of histology and these three proteins through available data on TCGA, and demonstrated increased expression of *NTRK2* and *CHEK2* with gene expression profiling. Based on the provocative results for enhanced TrkB expression in resected SCC tumors, as well as prior evidence suggesting its role in tumor progression, we then performed *in vitro* and *in vivo* experiments, including invasiveness and cell survival assays, to determine whether there was a suggestion of superiority in combined EGFR and TrkB inhibition compared to either of these treatments alone. Our results indicated that the effects were cell-line specific and not generalizable to a histologic subtype, though it was notable that there was a suggestion of synergy in head and neck cancer cell lines as well.

There has been increasing interest in TrkB recently based on data showing its correlation with prognosis and tumor aggressiveness in multiple tumor types, including neuroblastoma, gastric cancer, and hepatocellular carcinoma. There have also been several studies suggesting an association with poor outcomes in lung cancer. For instance, 102 NSCLC specimens were evaluated by immunohistochemistry for TrkB, and TrkB-positive tumors had higher rates of disease free and overall survival [27]. A more recent analysis showed that TrkB inhibition led to a loss of vimentin and other transcription factors that are linked to the EMT transition [28]. Furthermore, it was recently reported that the wild-type, not mutant form, of TrkB enhanced cell migration and transformation, suggesting that mutations in this protein should not be used to select patients for treatment [39]. Rather, Trk fusions appear to be of greater import in treatment and prognosis. Fusions in TrkA, TrkB, and TrkC have been demonstrated in several tumor types [40], and heightened signaling through this mechanism has been shown to promote metastatic progression and lead to metastatic progression [41] and poorer prognosis [42]. Furthermore, it has recently been shown *in vitro* that delivering AZD7451 to colorectal and large cell neuroendocrine cells expressing NTRK1 and NTRK2 inhibited growth and proliferation, and that the presence of an NTRK1 fusion increased the antitumor effects of AZD7451. As a result of these recent studies, there has been a renewed interest in Trk inhibitors, with Trk fusions serving as a putative biomarker to select patients for treatment [43].

With regard to preclinical findings of TrkB as a viable target in lung cancer, a recent study showed that in lung adenocarcinoma models driven by KRAS and p53 loss, increased TrkB expression was correlated with more aggressive lung cancer tumors, and that TrkB was required for lung tumor metastases *in vivo*. These findings were supported by patient data showing that TrkB was associated with an increased rate of metastatic progression [44]. In contrast, the current analysis focused on SCC, and based on our findings in human tumor specimens that TrkB was elevated in SCC in two different tissue databases, in the latter half of the study we provided preclinical evidence of TrkB as a therapeutic target in SCC. By combining EGFR and TrkB tyrosine kinase inhibitors, we showed that treating certain cell lines with EGF led to increased TrkB activity, and that this effect could be attenuated by erlotinib. While this effect was not generalizable to all cell lines tested, the results suggest that in certain cells, the effect of inhibition of the EGFR and TrkB TKI pathways together was stronger than either alone.

There are several studies in other diseases suggesting crosstalk between the TrkB and EGFR receptors, implying a synergistic effect. In ovarian cancer cell lines, both EGF and BDNF can transactivate the receptors and activate Akt, a downstream target. Furthermore, EGFR and TrkB inhibitors impede EGFR activation, and both of these inhibitors, as well as PI3K/Akt inhibitors, reduce cell migration induced by both EGF and BDNF [35]. In NSCLC, TrkB expression enhances the ability of EGF to induce wound healing. In contrast, reduced TrkB expression is associated with substantial changes in the cytoskeleton and reduced E-cadherin expression, suggesting that loss of TrkB promotes cell migration [45]. Finally, in human colon cancer cells, cetuximab reduces the expression of both BDNF and TrkB, and this effect can be attenuated by the addition of recombinant BDNF [46]. In the current study, after observing the association of TrkB with SCC and the preferential effect on SCC cell lines of TrkB inhibition, we explored the crosstalk between TrkB and EGFR of several cell lines. We provide evidence of synergy between EGFR and TrkB inhibitors in the vast majority of adenocarcinoma cell lines, and in two SCC cell lines. It is notable that in two of the SCC cell lines that demonstrated apparent antagonism there was a marked effect of AZD7451 alone, which could have in turn masked a synergistic effect. However, in repeating both the migration/invasiveness and crosstalk assays on several additional cell lines, the results were again not uniform. Therefore, these phenomena also appear to be cell-line specific. The implication of these results is that combination therapy will be effective in a select group of patients with lung cancer, and while this initial data is promising given the development of EGFR resistance and lack of therapeutic options in the context of SCC, further refinement is necessary in defining appropriate patient subsets for this treatment.

This data thus expands on prior reports of the relationship between these receptors in other malignancies, and is the most comprehensive analysis in SCC lung cancer cell lines to our knowledge. Taken in the context of prior data, our data suggests that wild-type TrkB may be a therapeutic target for SCC, and that combination of TrkB blockade with an EGFR inhibitor may enhance the efficacy of both. Indeed, we acknowledge that sensitizing EGFR mutations are not commonly found in SCC lung cancers, and that these mutations are the strongest predictors of response to EGFR tyrosine kinase therapy. In addition, we examined several EGFR wild type cell adenocarcinoma cell lines as well, and again observed this effect (suggesting that it is not selective for SCC). We thus believe that this analysis provides compelling data regarding the potential combination of these two agents in both wild type adenocarcinoma and SCC of the lung, though we do acknowledge that the inconsistent effect among cell lines of the same histology does not clearly identify which patients may benefit from this combination treatment. The true mechanism behind this observation and the determination of which patients may benefit from this approach is the focus of future study.

We also found that DNA repair machinery was elevated in SCC tumors, including PARP, CHK2, and pCHK2. Indeed, CHK2 is a protein involved in the repair of DNA damage and cell cycle arrest, and Chk inhibitors have been suggested to have efficacy in improving response in multiple tumor types [47]. Chk inhibition has also been associated with radiosensitization, possibly through abrogation of the G2 checkpoint [48]. However, data regarding the efficacy of Chk inhibition in lung cancer is limited. In one analysis, it was found that radiosensitivity was heightened with a combination of celecoxib and gefitinib, and that this effect was at least partially mediated through the synergistic enhancement of RT-induced arrest, implying the role of Chk inhibition [49]. And in another recent study, it was shown that the Chk1/2 inhibitor AZD7762 enhanced responsiveness to chemotherapy both *in vitro* and *in vivo*, and that concurrent use of these agents led to a significant reduction of cancer stem cells in mouse xenografts [50]. Phase I testing of the Chk1 inhibitors is in progress, although cardiac toxicity was observed in a trial of one such agent AZD6672 [51]. Nevertheless, if effective inhibitors exhibit a tolerable safety profile, our data suggests further investigations in lung SCC may be merited.

Finally, we observed alterations in the oxidative stress pathway in SCC tumors. Specifically, we found that Keap1 and Nrf2 were both increased in SCC in both TCGA and the PROSPECT database, with Keap1 being the most highly correlated protein with SCC in PROSPECT. In the unaltered state this finding would be unexpected, since Keap1 has been found to repress levels of Nrf2, leading to its degradation [52, 53]. However, when one of these two genes is mutated, the level of interaction between the two proteins is reduced and therefore this inverse correlation may not be observed. Our finding that both proteins were increased in SCC is consistent with the TCGA analysis demonstrating significantly altered pathways with *NFE2L2* and *KEAP1* in 34% of patients [54], and suggests that the oxidative stress response is more pronounced in SCC.

It is important to note that while the primary focus of this paper was lung cancer, there are clear parallels between SCC histologic subtypes of the lung and other tissue types that both provide the justification for examining TrkB inhibition in H/N SCC cell lines, as well as potentially expanding these findings to other SCC tumors. For example, the comprehensive SCC TCGA analysis of H/N SCC similarly demonstrated alterations in multiple RTKs, such as EGFR and PIK3CA, as well as aberrations in the oxidative stress pathway that includes KEAP and NRF2 [55]. In addition, it has previously been observed, through an analysis of 12 tumor types, that molecular characteristics can be largely preserved by histologic subtype, even across primary site. That is, clustering of molecular subtypes is more similar between H/N SCC and lung SCC than between LUAD and lung SCC [38]. To this end, a “squamous cell” signature has previously been cited across head and neck, lung, cervical, and bladder cancers [36]. This squamous cancer subset was also identified through an RPPA analysis of 11 TCGA “Pan-Cancer” diseases, further supporting molecular preservation independent of tissue type [37]. It is for these reasons that TrkB inhibitors could be of use across tumor subtypes, specifically lung and H/N SCC, and our data supports further exploration in both clinical contexts.

One notable limitation of this paper is the absence of mechanistic data underlying the effects of TrkB, and indeed there is limited information in the literature on this subject. For example, the mechanism of TrkB induction is largely unknown, though several potential biological processes may occur, including gene amplification, mutations, or translocations, a question which can be explored in future analyses. Furthermore, the specific interactions that take place at a molecular level between TrkB and EGFR were beyond the scope of this manuscript. It is possible that TrkB is a resistance/escape mechanism for EGFR through downstream effects, such that the combination of these two agents is superior to an EGFR inhibitor alone. Or, EGFR and TrkB could be expressed preferentially in the same cells and in specific cell lines, thus leading to selective synergy. Future analyses will focus further on addressing these mechanistic questions at the preclinical level.

In conclusion, through RPPA analysis of surgically resected NSCLC tumors, we found increased expression of TrkB in SCC, a finding that complements prior data in the adenocarcinoma subtype and highlights the importance of TrkB fusions in prognosis. Our data support the use of TrkB as a targetable protein in subsets of SCC, and is strengthened by our preclinical evidence showing crosstalk between this receptor and EGFR, suggesting a novel treatment approach. In addition, we have shown enhanced expression of several proteins related to DNA repair machinery, such as Chk2, pChk2, and PARP, suggesting the potential co-targeting of this pathway in SCC. Finally, we found that both KEAP1 and NRF2 were elevated in SCC tumors, both in our institutional database as well as TCGA, providing further evidence of the role of the oxidative stress pathway in this histologic subtype.

## MATERIALS AND METHODS

### Reverse phase protein array analysis

We have described our RPPA technique in several prior publications.[9, 14] Briefly, frozen PROSPECT tumor tissue were added into cold RPPA buffer [1% Triton X-100, 50mM Hepes, PH 7.4, 150mM NaCl, 1.5mM MgCl_2_, 1mM EGTA, 100mM NaF, 10mM Na Pyrovate, 1mM Na_3_VO_4_, 1mM PMSF, 10% glycerol, containing protease inhibitors and phosphatase inhibitors] and then the tissues were homogenized by electric homogenizer (Power Gen 125, Fisher Scientific, Houston, TX). After centrifugation, the protein lysates were collected, and lysate concentration were determined. Normalization occurred with the same starting concentration of 12mg/ml, followed by combination with the 4 X SDS sample buffer without bromophenol blue. The samples were then serially diluted with 5 dilutions (1:2-1:16) with dilution buffer, which contained three parts lysis buffer and one part 4 X SDS sample buffer + 2-mercaptoethanol. 2 X Phosphate-buffered saline solution (Mg^2+^, Ca^2+^, free) containing 60% glycerol in equal amounts, was added to the well plates, and RPPA was then printed using Aushon 2470 Arreyer (Aushon Biosystems, Billerica, MA) and the analysis performed as described in our previous publications.[56]

For each array, antibody staining was done at room temperature and with Dako Autostainer Plus (Dako North America, Inc. Carpinteria, CA). The Dako Catalyzed Signal Amplification system was used to detect each signal, in accordance with the manufacturer's recommendations (DakoCytomation California, Inc., Carpinteria, CA). The list of antibodies and details pertaining to antibody optimization and validation has been described in previous studies.[9, 57] Notably, the antibodies utilized in the key signaling pathways underwent extensive validation with Western blots, during which band quality and association of protein levels with RPPA were determined, in accordance with prior analyses.[9] Finally, about ten percent of samples were produced in duplicate in serial dilutions on a single slide to assess regional variability within an array, which was then probed with an antibody that recognized a unique protein. The level of antibody binding was then quantified. This overall process reduces interassay variation by utilizing multiple replicates and thus increasing the robustness of comparisons between samples.

### RPPA processing and statistical analysis

We used MicroVigene software (VigeneTech, Carlisle, MA) and an in-house R-package [58] to measure spot intensity. Quantification was performed utilizing a SuperCurve method which uses MicroVigene software (VigeneTech, Carlisle, MA) to determine changes in protein level, as well as an R package that was developed at our institution [58]. This technique produces a logistic curve by pooling the intensity data of each spot from all samples on each slide. Then, the dilution specifications are mapped on a SuperCurve, so that quantification can be achieved. We utilized median-control normalization for each analysis, and utilizing R, version 2.7.0.

We adjusted for differences in sample loading by using a “whole antibody set” approach, which is available as a script in the R package. Duplicate samples as described above were averaged for the purpose of analysis, and two-sample *t-*tests were used to compare protein levels between the subgroups of interest in this study, specifically SCC vs. non-SCC histology. To adjust for multiple comparisons, we applied a beta-uniform mixture (BUM) to model the resulting p-values computed from the test statistic, and a false detection rate (FDR) of 1% was used as a cutoff to identify significant differences between patient subgroups [59].

### Validation of PROSPECT findings using TCGA

The Cancer Genome Atlas (TCGA) is a National Cancer Institute-funded effort to sequence and analyze a wide variety of malignancies through the contribution of tissue by several institutions nationally. Genomic characterization of SCC of the lung was previously performed using 178 tumors from this source [54]. For this analysis, we utilized the tumors in our prior report and other specimens for which there was RPPA data available, for a total of 195 patients with SCC. The adenocarcinoma subset included patients from the TCGA website for which RPPA data analysis was available (n=181).

### Expression profiling

Our gene expression data have been archived at the Gene Expression Omnibus repository (GSE42127) and have been reported elsewhere [17, 60–63]. To determine if those proteins that had were increased in SCC on RPPA also demonstrated increased expression at the mRNA level, we performed gene expression analysis on selective, potentially targetable genes that met this criterion. To do so, RNA was isolated from PROSPECT tumors by Tri Reagent and column-based purification (Qiagen). Profiling was then done using the Illumina WGv2 and Illumina WGv3 arrays (Illumina). Optimal probes were selected based on multiple criteria, including: 1) targeting spliced region on gene, 2) proximity to 3’ end, and 3) preservation in other mammals.

We then attempted to validate the gene expression results using the TCGA database, again focusing on the proteins that were preferentially expressed in SCC using RPPA. To assess for differences between lung adenocarcinoma, lung SCC, and head/neck SCC, we utilized the TCGA pan-cancer database that has been previously reported [64]. Finally, to further elucidate the reliability of gene expression profiling, we utilized the genome browser to determine the strength of each individual probe. Several criteria were used to determine on which probes the most emphasis should be placed, including: 1) location near the 3’end, 2) inclusion in the spliced region, 3) preservation in other homologs of the same gene, and 4) preservation in other animals. This analysis was focused on those probes that gave inconsistent results in initial profiling (e.g. association with one probe but not another for the same gene).

### Detection of TrkB

Tumor cell protein lysates were collected, and TrkB and phosphorylated TrkB levels were evaluated by ELISA (Cell Signaling, Danvers, MA) using 200 μg protein and according to manufacturer's instructions. For Western blotting, tumor cells were serum starved for 24 hours and then treated with 50ng/ml of BDNF (R&D Systems) for 15 minutes or AZD7451 (1μM) for 1 hour. Protein lysates were collected, separated by SDS PAGE, and blotted onto a nitrocellulose membrane. Membranes were incubated with antibodies directed against TrkB, p-TrkB, pErk, and pAKT (all 1:1000; Cell Signaling), and then incubated with appropriate secondary antibodies. Bands were visualized using ECL.

### Drug sensitivity of cell lines

Cell lines were plated at a density of 2,000 cells per well in each well of a 96 well plate. After a 24h incubation period, cells were treated with increasing concentrations of erlotinib or AZD7451. After 5 days cell viability was measured by MTS assay. IC50 values were log transformed (base 10) prior to analysis. Differences in the relative IC50s (the percentile (0-100%) of each cell line's IC50 across all cell lines tested) were compared between SCC and non-SCC cell lines with the Wilcoxon rank sum test. We selected agents for this comparison that targeted proteins found to have increased expression on RPPA. The Chou-Talalay method implemented by the drexplorer software was used to estimate the interaction index (IAI) to assess the degree of drug interactions for combination treatments. IAI values less than 1 indicate a trend towards synergism and IAI values larger than 1 indicate a trend towards antagonism. Values within 0.05 of the value 1 were labeled “additive.”

### Migration assay

700μL of serum-free RPMI containing BDNF, NT3, or NGF (50ng/mL; R&D Systems) alone or in combination with AZD7451 (1μM) was added to the lower compartment of 24-well polycarbonate Transwell migration inserts (8.0μm pore size; Fisher Scientific, Pittsburgh, PA). Tumor cells cells (5×10^4^) were added to the upper chambers and incubated for 20 hours. Cells in the upper compartment were removed. Cells that migrated to the underside of the membrane were stained and counted.

### Animals and tumor xenografts

Male athymic nude mice (NCI-nu) were obtained from the Animal Production Area of the National Cancer Institute (Frederick Cancer Center, Frederick, MD). The mice were housed and maintained under pathogen-free conditions in facilities approved by the American Association for Accreditation Laboratory Animal Care and in accordance with current regulations and standards of the U. S Department of Agriculture, the U. S. Department of Health and Human Services, and the National Institutes of Health. The mice were used, in accordance with institutional guidelines, when they were 6 to 8 weeks old. To generate tumor xenografts, we harvested HCC95 tumor cells from subconfluent cultures by briefly exposing the cells to asolution containing 0.25% trypsin and 0.02% EDTA (Life Technologies). Cells were washed twice with serum–free medium, resuspended in Hanks’ balanced salt solution (HBSS), and HCC95 cells (2.0 × 10^6^) in 100 μl were injected into the subcutaneous flank of mice. Body weights and tumor volumes were recorded twice weekly. When the average of tumor volume had reached approximately 200 mm^3^, mice randomized into one of four treatment groups: (a) control; (b) oral administration of erlotinib (100 mg/kg) daily p.o.; (c) oral administration of AZD7451 (20 mg/kg) daily and (d) erlotinib plus AZD7451.

When assessing for differences in growth curves, the data included tumor size measurements at different times so that we have to consider the correlations between the measurements on the same mouse. We fit linear model (with treatment, time effect, and its interaction effect) using the generalized least squares method. We used the Akaike information criterion (AIC) for selecting the best correlation structure. Tukey's HSD (honest significant difference) test is used for post-hoc pairwise comparison. The analysis was performed using nlme and multcomp packages in R.

### Statement of significance

Through a proteomic analysis of lung squamous cell carcinoma (SCC) we identified multiple therapeutic targets including TrkB, which promoted tumor cell invasivenessand demonstrated synergistic effects when combined with an EGFR inhibitor in cell-line specific adenocarcinoma and SCC.

Dr. Heymach would like to acknowledge the following funding sources: The University of Texas Southwestern Medical Center and The University of Texas MD Anderson Cancer Center Lung SPORE grant 5 P50 CA070907; DoD PROSPECT grant W81XWH-07-1-0306; Lung Cancer Moon Shot Program; National Institute of Health Cancer Center Support Grant (CA016672); R01 CA168484; R01CA190628; David Bruton, Jr. Endowed Chair to J.V.H; Rexanna Foundation for Fighting Lung Cancer.

## SUPPLEMENTARY MATERIALS FIGURES AND TABLES




