# Supplementary material for: Integrative proteomic and transcriptomic analysis provides evidence for TrkB (NTRK2) as a therapeutic target in combination with tyrosine kinase inhibitors for non-small cell lung cancer
Source: Oncotarget. 2018 Jan 30;9(18):14268–84. doi: 10.18632/oncotarget.24361 (PMC5865668; doi:10.18632/oncotarget.24361)
Supplement: Supplementary file 1 [file oncotarget-09-14268-s001.pdf]

Integrative proteomic and transcriptomic analysis provides evidence for TrkB (NTRK2) as a therapeutic target in combination with tyrosine kinase inhibitors for non-small cell lung cancer

SUPPLEMENTARY MATERIALS

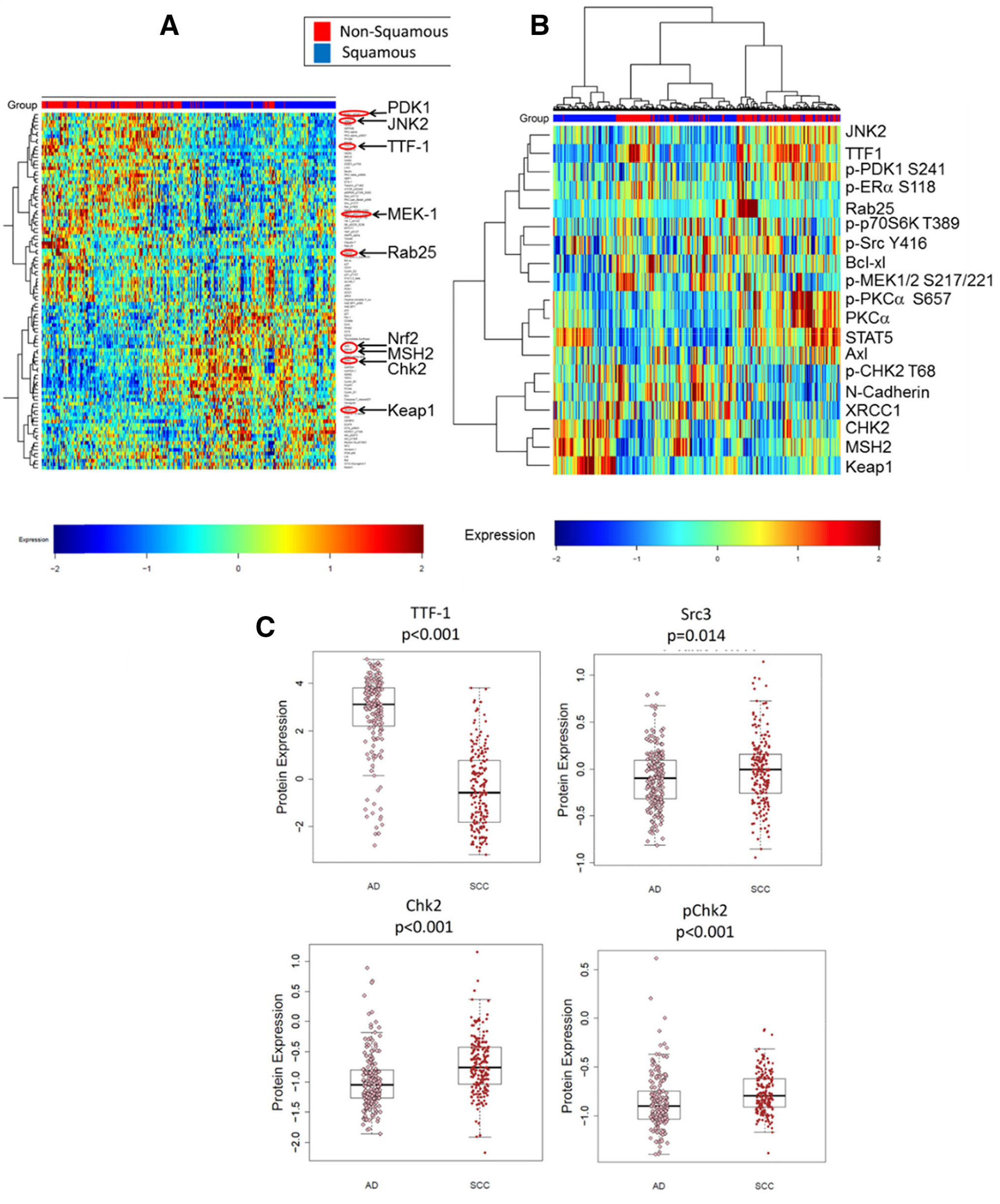

Supplementary Figure 1: TCGA validation of PROSPECT findings. (A) Hierarchical clustering heat map with 100 proteins that achieved the highest level of significance, with some key proteins highlighted, **(B)** Heat map of protein levels by histology of top 29 hits in Figure 1A that were also tested in TCGA. Several proteins demonstrated the same variations by histology in both datasets, including JNK2, TTF-1, PDK1, and Rab25. **(C)** TCGA validation demonstrates increased levels of Src3, Chk2, and pChk2 in SCC tumors, concordant with the findings in the PROSPECT database.

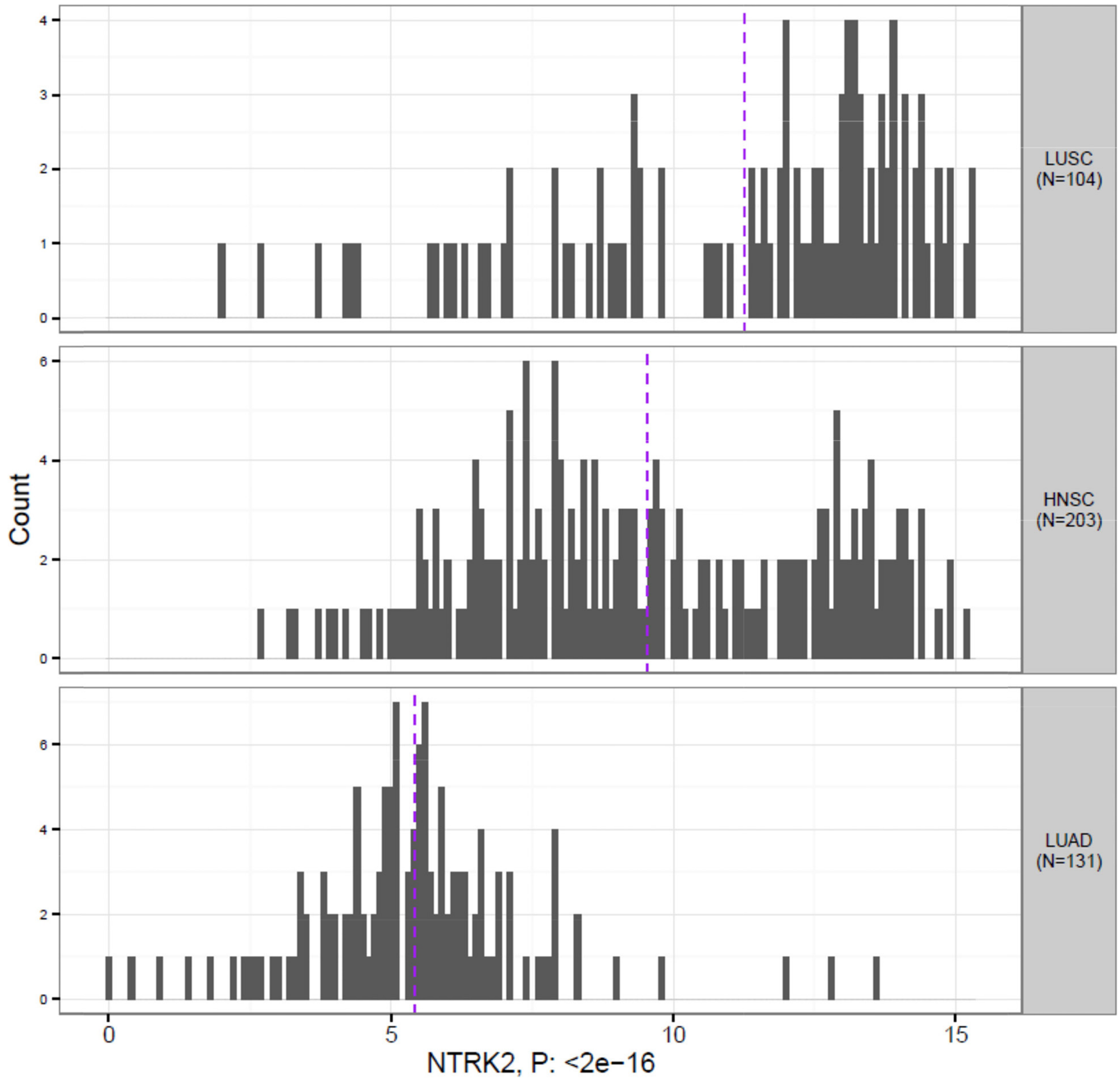

Supplementary Figure 2: Probe analysis demonstrating that expression profiles were more similar between lung and headn/neck SCC than with adenocarcinoma and lung SCC.

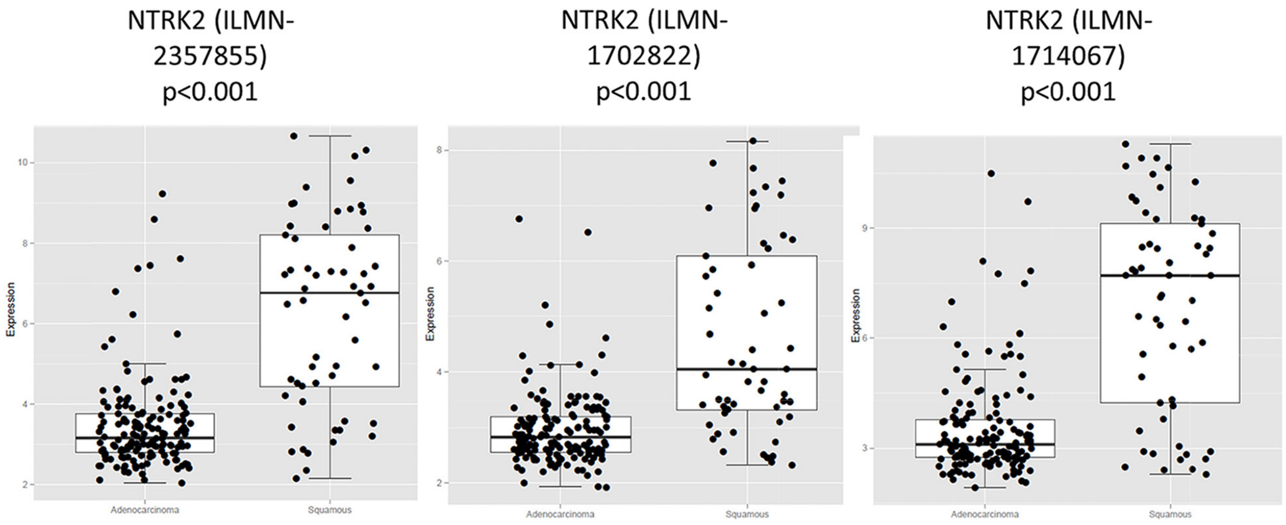

Supplementary Figure 3: Variations in *NTRK2* expression by histology with all probes used in this analysis.

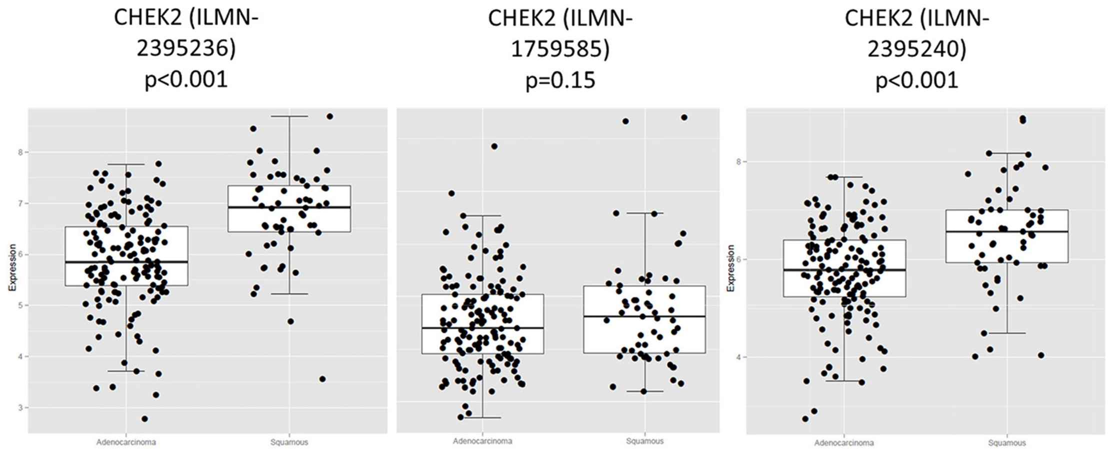

Supplementary Figure 4: Variations in *CHEK2* expression by histology with all probes used in this analysis.

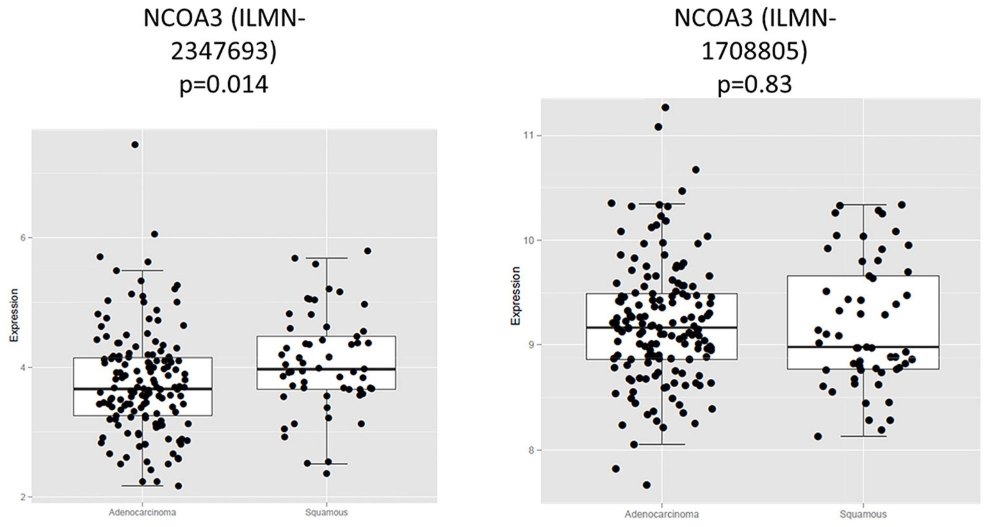

Supplementary Figure 5: Variations in *NCOA3* expression by histology with all probes used in this analysis.

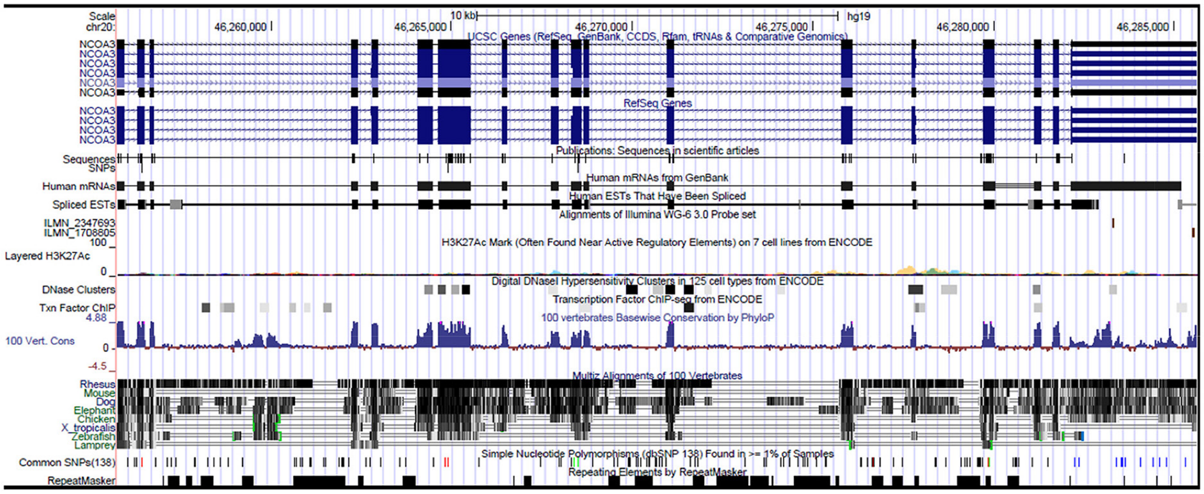

Supplementary Figure 6: Genome browser utilized in gene expression analysis of NCOA3. The gene for ILMN-2347693 demonstrated increased expression in SCC but was located in a non-spliced region.

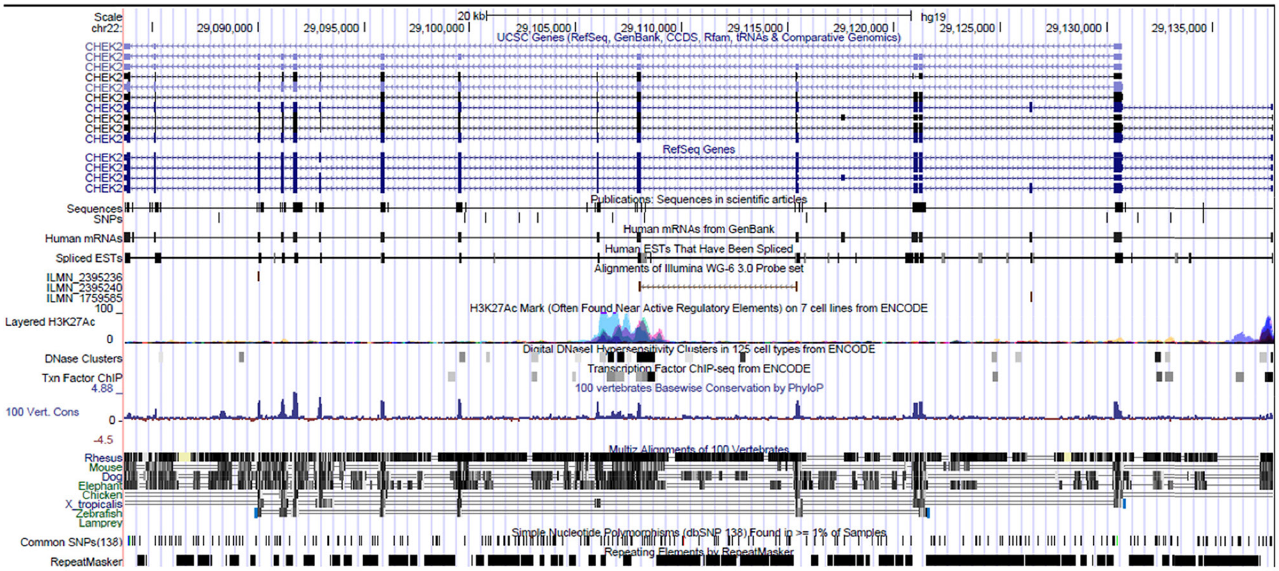

Supplementary Figure 7: Genome browser utilized in gene expression analysis of CHEK2. Two of the three probes demonstrated increased expression in SCC, and the gene for the probe that did not (ILMN-1759585) was located distant from the 3' region and is not preserved in other animals.

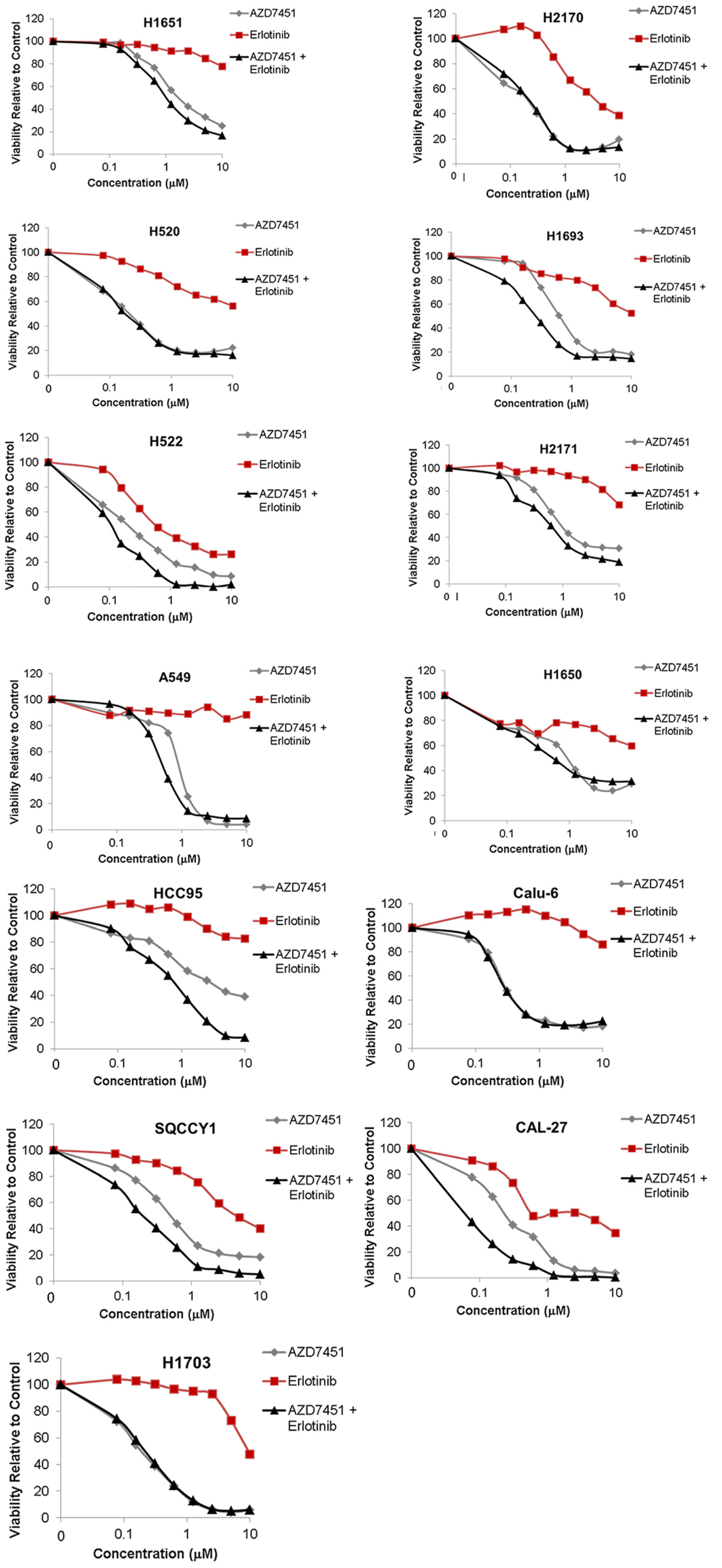

Supplementary Figure 8: Growth curves demonstrating inhibition with erlotinib, AZD7451, and combination therapy.

Supplementary Table 1: Patient characteristics of datasets in study

Characteristic	PROSPECT n (%) (total n=140)	TCGA SCC n (%) (total n=195)	TCGA non-SCC n (%) (total n=181)
Female	59 (42)	40 (21)	105 (58)
Male	81 (58)	115 (59)	76 (42)
Unknown	0 (0)	40 (20)	0 (0)
Squamous Cell Carcinoma	34 (24)	195 (100)	0 (0)
Non-SCC*	106 (76)	0 (0)	181 (100)
Stage I	78 (56)	80 (41)	95 (52)
Stage II	24 (18)	39 (20)	39 (22)
Stage III	36 (26)	33 (17)	40 (22)
Stage IV	1 (<1)	43 (22)	7 (4)
Unknown	0 (0)		
Smoking			
No	12 (9)	10 (5)	27 (15)
Yes	128 (91)	140 (72)	147 (81)
Unknown	0 (0)	45 (23)	7 (4)
For adenocarcinoma patients,			
KRAS			
Mutant	35 (33)	N/A	36 (24)
Non-Mutant	71 (67)		134 (74)
Unknown	0 (0)		0 (0)
For adenocarcinoma patients,			
EGFR			
Mutant	12 (11)	N/A	27 (15)
Non-mutant	94 (89)		154 (85)
Unknown	0 (0)		0 (0)

Supplementary Table 2: Full list of proteins differentially expressed in SCC vs. non-SCC (listed by significance value)

See Supplementary File 1

Supplementary Table 3: Crosstalk between EGFR and TrkB inhibition in both SCC and adenocarcinoma cell lines, demonstrating synergy in all but one head and neck cell line, and two SCC cell lines

Cell Line	SCC vs. Adenocarcinoma	AZD7451 IC_{50} (μm)	Erlotinib IC_{50} (μm)	Chou Talalay Interaction Index (IAI)	Synergy/ Antagonism (IAI<1=Synergy, IAI>1=Antagonism)
H520	SCC	0.19	8.51	1.01	Additive
H2170	SCC	0.17	5.88	1.42	Antagonism
HCC95	SCC	3.24	7.8	0.21	Synergy
H1703	SCC	0.15	5.2	1.13	Antagonism
SQCCY1 (Head and Neck SCC)	SCC	0.6	4.86	0.34	Synergy
Cal-27	SCC	0.24	2.6	0.16	Synergy
H1693	Adenocarcinoma	0.7	14.1	0.27	Synergy
H522	Adenocarcinoma	0.17	0.85	0.48	Synergy
H2172	Adenocarcinoma	1.75	24.9	0.49	Synergy
H1651	Adenocarcinoma	2.33	>30	0.59	Synergy
A549	Adenocarcinoma	0.6	>30	<0.01	Synergy
Calu-6	Adenocarcinoma	0.5	11.73	1.04	Additive
H1650	Adenocarcinoma	0.85	>30	0.84	Synergy

Note that the two cell lines in which antagonism was observed were found to have strong effects with AZD7451 alone.
