# Supplementary material for: Integrative proteomic and transcriptomic analysis provides evidence for TrkB (NTRK2) as a therapeutic target in combination with tyrosine kinase inhibitors for non-small cell lung cancer
Source: Oncotarget. 2018 Jan 30;9(18):14268–84. doi: 10.18632/oncotarget.24361 (PMC5865668; doi:10.18632/oncotarget.24361)
Supplement: Supplementary file 2 [file oncotarget-09-14268-s002.docx]

Supplementary Table 2:  Full list of proteins differentially expressed in SCC vs. non-SCC (listed by significance value)

| Protein Marker | Effect in TCGA (S=same, O=opposite, N=no effect, NT=not tested) | Difference (fold-change) | T Score | p-value |
| --- | --- | --- | --- | --- |
| **Increased in SCC** |  |  |  |  |
| Keap1 | S | 1.64 | 6.75 | <0.001 |
| TrkB | NT | 1.56 | 5.34 | <0.001 |
| CHK2 | S | 1.40 | 5.08 | <0.001 |
| pRb.S807 | NE (trend, p=0.20) | 1.34 | 5.06 | <0.001 |
| pCHK2.T68 | S | 1.28 | 5.05 | <0.001 |
| IGF1R | NT | 1.23 | 4.14 | <0.001 |
| MSH2 | S | 1.28 | 3.50 | <0.001 |
| Src3 | NT | 1.12 | 2.84 | 0.005 |
| CA-9 | S | 1.27 | 2.31 | 0.022 |
| Cyclin B1 | S | 1.31 | 2.27 | 0.025 |
| Cleaved PARP | NT | 1.37 | 2.25 | 0.026 |
| Nrf2 | S | 1.10 | 2.13 | 0.035 |
| PI3K.p110 | S | 1.10 | 2.08 | 0.039 |
| Cyclin E1 | S | 1.17 | 2.08 | 0.039 |
| **Decreased in SCC** |  |  |  |  |
| TTF-1 | S | -3.41 | -7.17 | <0.001 |
| pMEK1.S217 | S | -1.14 | -4.43 | <0.001 |
| pER | NT | -1.21 | -4.12 | <0.001 |
| pPDK1 | S | -1.18 | -4.08 | <0.001 |
| pPKCα | S | -1.26 | -3.95 | <0.001 |
| Met | S | -1.18 | -3.81 | <0.001 |
| Rab25 | S | -1.28 | -3.76 | <0.001 |
| p70S6K.T389 | S | -1.15 | -3.66 | <0.001 |
| PKCa | S | -1.34 | -3.36 | 0.001 |
| PTCH | NT | -1.18 | -3.34 | 0.001 |
| Bcl.xL | S | -1.12 | -3.22 | 0.002 |
| JNK2 | S | -1.12 | -3.21 | 0.002 |
| N-cadherin | O | -1.13 | -3.19 | 0.002 |
| XRCC1 | O | -1.09 | -2.95 | 0.004 |
| BRF2 | NT | -1.10 | -2.91 | 0.004 |
| Stat5 | NE (trend, p=0.21) | -1.30 | -2.86 | 0.005 |
| pEGFR.Y992 | NT | -1.11 | -2.82 | 0.005 |
| Notch3 | NT | -1.12 | -2.82 | 0.005 |
| Axl | O | -1.13 | -2.75 | 0.007 |
| pSrc.Y416 | NE (trend, p=0.113) | -1.18 | -2.75 | 0.007 |
| FGFR1 | NT | -1.14 | -2.67 | 0.008 |
| pStat3.Y705 | S | -1.21 | -2.65 | 0.009 |
| FANCD2 | NT | -1.15 | -2.61 | 0.010 |
| DTYMK | NT | -1.15 | -2.56 | 0.012 |
| p21 | O | -1.13 | -2.55 | 0.012 |
| pMet | NT | -1.08 | -2.44 | 0.016 |
| MACC1 | NE (trend, p=0.055) | -1.26 | -2.42 | 0.017 |
| Cyclin D1 | NE (p=0.66) | -1.11 | -2.41 | 0.017 |
| FAK | NT | -1.34 | -2.34 | 0.021 |
| CD31 | S | -1.13 | -2.28 | 0.024 |
| COX2 | NT | -1.22 | -2.27 | 0.025 |
| pHER2.Y1248 | NE (trend, p=0.095) | -1.08 | -2.19 | 0.030 |
| p16.INK4A | O | -1.11 | -2.16 | 0.032 |
| p4EBP1.T70 | O | -1.08 | -2.16 | 0.032 |
| cKit | S | -1.31 | -2.14 | 0.034 |
| p90RSK | S | -1.09 | -2.09 | 0.038 |
| pStat5 | NT | -1.10 | -2.04 | 0.043 |
| PI3Kp85 | O | -1.09 | -2.04 | 0.044 |
| mTOR | NE | -1.08 | -2.03 | 0.044 |
